# SGLT2 inhibitors and mortality in diabetic cancer patients: cardioprotective and anticancer effects

**DOI:** 10.1093/ehjcvp/pvaf044

**Published:** 2025-06-06

**Authors:** Kalliopi Keramida, Ali Ahmed, Gerasimos Filippatos

**Affiliations:** Department of Cardiology, General Anticancer Oncological Hospital, Agios Savvas, Athens 11522, Greece; Department of Medicine, Veterans Affairs Medical Center, Washington, DC 20422, USA; Department of Medicine, Georgetown University, Washington, DC 20007, USA; Department of Medicine, George Washington University, Washington, DC 20052, USA; Department of Cardiology, Attikon University Hospital, National and Kapodistrian University of Athens, School of Medicine, Athens 12462, Greece

**This editorial refers to ‘Effects of sodium–glucose cotransporter 2 inhibitors in patients with cancer and diabetes mellitus: a systematic review and meta-analysis’, by G. Novo *et al*., https://doi.org/10.1093/ehjcvp/pvaf028.**

**This editorial refers to ‘SGLT-2 inhibitors in cancer patients with diabetes: when cardioprotection is key’, by M. Brambilla *et al*., https://doi.org/10.1093/ehjcvp/pvaf038.**

Patients with Diabetes Mellitus Type 2 (DM2) are known to have a higher risk of cardiovascular (CV) complications. However, these patients are also at a higher risk of developing several neoplasms, the treatment of which adds to the burden of CV complications. This cardiotoxicity, especially when severe, significantly impacts morbidity and mortality of cancer patients and survivors. Several pre-clinical and clinical studies of beta-blockers, angiotensin-converting enzyme inhibitors, angiotensin receptor blockers, and statins have generated mixed results in lowering the risk of chemotherapy-associated cardiotoxicity. However, sodium-glucose cotransporter-2 inhibitors (SGLT2Is) have shown in meta-analyses consistent effectiveness to lower the risk of death and heart failure hospitalizations (HFHs).^1–3^

**Figure 1:**
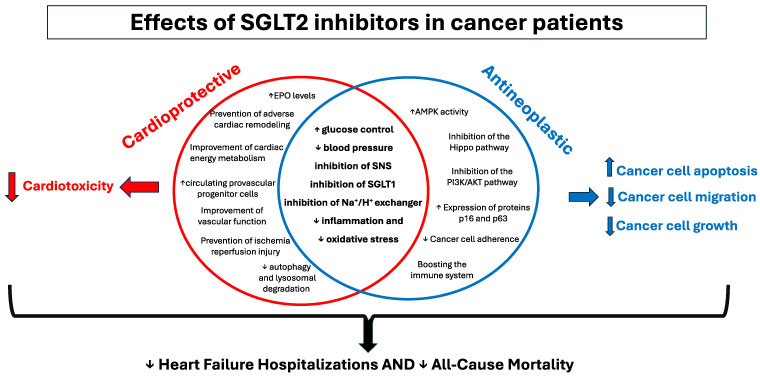
Cardioprotective and antineoplastic effects of sodium-glucose cotransporter-2 inhibitors.

In this issue of the Journal, Novo et al. reported the results of a meta-analysis of 11 observational studies of >100 000 patients with DM2 and cancer receiving antineoplastic therapies and compared CV outcomes between those receiving SGLT2Is vs. other antidiabetic medications.^4^ The authors reported that patients in the SGLT2Is group had a 53% associated lower risk of death and a 56% lower associated risk of HFHs. Respective lower associated risks for anthracyclines were 49% and 75%. These findings are consistent with those from previous meta-analyses,^1–3^ but this meta-analysis includes a larger number of patients and is the only one including a subgroup analysis of diabetic patients, with and without baseline heart failure (HF). Although the magnitudes of the risk reduction in these observational studies are likely inflated by confounding and prevalent user bias, and these findings need to be confirmed in randomized controlled trials, these findings are mechanistically plausible due to the known cardioprotective and antineoplastic properties of SGLT2Is. SGLT2 is localized at the proximal tubule in the kidneys and is responsible for reabsorption of 80%–90% of the glucose filtered by the kidney. However, functional expression of SGLT2 in a wide variety of tumours and direct inhibition of glucose uptake in cancer cells by SGLT2Is suggest that these are potential therapeutic targets. Several studies have shown the anticancer effects of SGLT2Is, i.e. increased cancer cell apoptosis and decreased cancer cell growth and migration (*Figure 1*).^5^ SGLT2Is increase the activity of adenosine monophosphate-activated protein kinase (AMPK), inhibit the Hippo and PI3K/AKT/mTOR pathways, enhance the expression of proteins p16 and p63, boost the immune system, and reduce cancer cells adhesion—all of which are crucial factors in tumours’ growth and cancer progression.^6^

AMPK modulates inflammatory and metabolic pathways to suppress tumour development and progression. SGLT2Is can indirectly activate AMPK in cancer cells leading to cell arrest and induction of apoptosis and ferroptosis.^7^ Hippo signalling pathway plays an important role in regulating organ size, embryonic development, tumourigenesis, and self-renewal of stem cells. Any disruption of Hippo pathway can result in uncontrolled cell growth and malignant transformation. Canagliflozin inhibits the Hippo signalling pathway by down-regulating the heterogeneous nuclear ribonucleoproteinK/YES-associated protein 1 axis in pancreatic cancer.^8^

PI3K/AKT/mTOR signalling pathway is implicated in cell growth, angiogenesis and survival. It is usually overactive in cancer, making it a promising target for therapeutic interventions. Everolimus, sirolimus, and duvelisib are some of the commercially available targeted inhibitors for cancer patients with abnormal activation of PI3K/AKT/mTOR pathway. Pre-clinical studies show that SGLT2Is inhibit this pathway in cancer cells leading to reduced cellular proliferation.^9^

p16 is a tumour suppressor protein that plays an important role in cell cycle regulation. By inhibiting CDK4/6 and CDK2, p16 prevents the phosphorylation of the retinoblastoma protein, leading to cell cycle arrest in the G1 phase. This halts the proliferation of cancer cells.^10^ Similarly, p63 regulates cell proliferation and apoptosis. Its up-regulation can lead to the activation of genes involved in cell cycle inhibition and programmed cell death, thereby suppressing tumour progression. SGLT2Is increase the expression of both p16 and p63 inducing cell cycle arrest and promoting apoptosis in cancer cells.^11^

The cardioprotective effects of SGLT2Is have been shown by large-scale clinical trials in diabetic patients. This drug class reduces the risk of CV events, by multiple mechanisms.^6^ Some cardioprotective effects can be considered antineoplastic too, namely lowering plasma glucose levels, reduction in blood pressure, suppression of sodium-hydrogen exchanger (NHE) activity, and mitigation of inflammation and oxidative stress (*Figure 1*).^12^

Glucose acts as the primary source of energy for cells, so hyperglycaemia and the cellular alterations induced may promote tumourigenesis. Optimal control of DM2 improves not only CV but also cancer outcomes, as it reduces cardiotoxicity, risk of cancer progression and enhances the antiproliferative effect of chemotherapy. Furthermore, SGLT2Is decrease insulin levels which acts as a growth factor that can promote cell proliferation and inhibit apoptosis.^13^

NHEs are ubiquitously expressed membrane transporters, with 11 human isoforms identified. Particularly the NHE-1 isoform plays a crucial role in regulating intracellular pH and extracellular acidification, both of which are fundamental to tumour growth, invasion, and metastasis, identifying it as a potential therapeutic target in cancer patients. Empagliflozin directly inhibits NHE1 activity as an off-target effect in human cardiomyocytes.^14^ Research is needed to explore SGLT2Is activity on NHE1 in cancer cells, potentially explaining part of their anticancer properties.

Finally, inflammation plays an important role in the pathogenesis and progression of cancer. SGLT2Is exert anti-inflammatory effects, which may explain a part of their beneficial effects. These combined effects not only protect the heart and the kidneys but may also reduce cancer progression by decreasing the pro-tumourigenic inflammatory milieu.

SGLT1 is also overexpressed in several cancers and is associated with enhanced tumour growth and poor prognosis. Inhibition of SGLT1 may confer a significant antineoplastic effect reducing glucose uptake in cancer cells and decreasing resistance to Epidermal Growth Factor Receptor Tyrosine Kinase Inhibitors.^15^ Some SGLT2Is have inhibitory effects on SGLT1, but data is limited.

In conclusion, SGLT2Is exhibit a multifaceted therapeutic profile, offering substantial cardioprotective and antineoplastic benefits to cardio-oncological patients. By mitigating inflammation, reducing oxidative stress, improving metabolic parameters, and modulating key signalling pathways involved in both CV disease and cancer, SGLT2Is hold the potential to improve outcomes in this complex patient population. The cardioprotective effects of SGLT2Is have been demonstrated in multiple randomized controlled trials in patients with HF, CV disease, DM2, and chronic kidney disease. Future prospective randomized controlled trials are needed to explore the cardioprotective and antineoplastic effects of SGLT2Is in patients with cancer receiving potentially cardiotoxic anticancer therapy.
